# Endothelium glutamate receptors in brain pathology

**DOI:** 10.3389/fphar.2025.1709274

**Published:** 2025-12-04

**Authors:** Sergey V. Pirozhkov, Marina N. Vukolova, Varvara V. Bulgakova, Yulia A. Lutokhina, Sergey B. Bolevich, Alexander I. Sobolevsky, Maria V. Yelshanskaya

**Affiliations:** 1 Department of Pathological physiology, Sechenov First Moscow State Medical University (Sechenov University), Moscow, Russia; 2 The Institute of Clinical Medicine named after N.V. Sklifosovskiy, Sechenov First Moscow State Medical University (Sechenov University), Moscow, Russia; 3 Faculty Therapy department №1, Sechenov First Moscow State Medical University (Sechenov University), Moscow, Russia; 4 Department of Biochemistry and Molecular Biophysics, Columbia University, New York, NY, United States

**Keywords:** glutamate receptors, blood-brain barrier, brain microvascular endothelial cell, ischemic brain injury, neuroinflammation, Alzheimer’s disease, systemic lupus erythematosus, multiple sclerosis

## Abstract

The endothelium in brain microcirculation functions not only as a barrier but also as a signal transduction component within a system that regulates multiple vascular processes, including muscle tone, permeability, and structural integrity. The control of local blood flow is vital to ensure adequate oxygen and nutrient supply, efficient removal of catabolic waste, and the maintenance of proper brain cell function. The role of endothelial glutamate receptors in brain pathology is an emerging area of research, particularly important for understanding how these receptors contribute to neurological diseases and disorders. Endothelial cells (ECs), which are considered active players in maintaining brain homeostasis, express glutamate receptors on their surface. Activation of these receptors can trigger a cascade of signaling events, including synthesis of nitric oxide (NO) and proinflammatory molecules. N-Methyl-D-Aspartate receptors (NMDARs) play a significant role in functional hyperemia, also known as neurovascular coupling (NVC), which is essential for maintaining the energy balance in brain cells. Growing evidence suggests that disturbance of this balance is implicated in several neurological diseases, such as Alzheimer’s disease, stroke, and traumatic brain injury (TBI), where endothelial dysfunction may impair blood flow regulation, contributing to further neuronal damage and cognitive decline. This review focuses on the glutamate receptor-mediated alterations in endothelial permeability and the prevention of the brain pathology through direct modulation of these receptors. Notably, the metabotropic glutamate receptor mGluR1, along with NMDARs, may cause deleterious effects in brain ischemia, as their activation increases the permeability of the vessel wall. Stimulation of NMDARs may also lead to ferroptosis in ECs. EC dysfunction results in significant blood-brain barrier (BBB) disruption, allowing infiltration by inflammatory cells and the accumulation in brain of pathological proteins, such as amyloid-beta (A_β_) or autoantibodies. This contributes to neuronal dystrophy and apoptosis, as seen in Alzheimer’s disease and autoimmune encephalopathy. Activated ECs generate proinflammatory mediators that attract leukocytes and sustain the neuroinflammatory response. Infiltrating peripheral white blood cells are key contributors to inflammatory damage following TBI. Regulation of ECs through glutamate receptors therefore represents a promising therapeutic strategy for treatment of neurodegenerative diseases, as well as ischemic and traumatic brain injuries.

## Introduction

Structures of the central nervous system are separated from blood by the blood–brain barrier (BBB) that controls the supply of necessary substrates, maintains ions’ homeostasis and protects brain from potentially toxic substances. Components of the BBB fine tune the cerebral blood flow in accordance with the metabolic demands of brain cells and their functional activity (Daneman and Prat, 2015). The BBB is constituted by layers of glycocalyx, endothelial cells (ECs), endothelial and parenchymal basement membranes, pericytes sitting in the duplication of the basement membrane, and astrocytic end-feet. BBB is also supported by microglia and neurons. This specialized complex of cells has been designated as “neurovascular unit”. The ECs of the BBB possess unique structural and functional characteristics that distinguish them from ECs in other tissues. These features include an increased number of mitochondria, absence of fenestrations, low pinocytotic activity, unique receptor-mediated endocytosis, and high abundance of tight junction proteins (such as claudins, occludins, and members of the junctional adhesion molecule family) (Bicker et al., 2014; Knowland et al., 2014). Additionally, adherence junction proteins (e.g., cadherins) and accessory cytoplasmic proteins (such as zonula occludens-1 and actin) contribute to the regulation of permeability. Toxic substances, including certain drugs that enter BBB ECs, are subjects of metabolic transformation by enzymes, such as cytochrome P_450_, monoamine oxidase, and others (Minn et al., 1991).

To ensure that brain regions with higher neuronal activity receive an adequate supply of blood, blood vessels constrict or dilate in the process of neurovascular coupling (NVC). NVC involves mechanisms initiated by neurons and contributed by various cell types (astrocytes, endothelial cells, pericytes) in the brain, ultimately resulting in the dilation of local arterioles or capillaries (Iadecola, 2017). ECs within these arterioles respond to several stimuli, including decreased partial pressure of O_2_, vasodilatory mediators, shear stress, and elaborate factors (NO, arachidonic acid metabolites) that cause relaxation of the smooth muscle cells in the vessel wall.

Significant portion of hemodynamic responses in the brain are regulated by endothelial NMDARs (Hogan-Cann et al., 2019). NMDARs are heterotetrameric non-selective cation channels that are formed by multiple subunits: GluN1, GluN2A, GluN2B, GluN2C, GluN2D, GluN3A, and GluN3B (Alexander et al., 2021; Hansen et al., 2018). Neuronal NMDARs are composed of two obligatory GluN1 subunits and either two GluN2 subunits or a mixture of GluN2 and GluN3 subunits (Negri et al., 2021; Hogan-Cann and Anderson, 2016). In addition, there are eight isoforms of GluN1 (GluN1-1a-GluN1-4a and GluN1-1b-GluN1-4b) resulting from alternative splicing (Paoletti et al., 2013). The subunit composition of NMDARs determines their functional properties. The canonical NMDAR is built of two GluN1 subunits that bind glycine and two GluN2 subunits that bind glutamate. GluN1 is involved in surface trafficking and anchoring of NMDARs at synaptic sites (Hansen et al., 2018). Activation of NMDAR requires simultaneous binding of two types of amino acids, glutamate to GluN2 and co-agonist glycine or D-serine to GluN1 (Traynelis et al., 2010).

Glutamate binds to post-synaptic NMDARs triggering the influx of extracellular Ca^2+^ and stimulating neuronal nitric oxide synthase to produce NO, thereby facilitating NVC. In addition to their neuronal localization, they are also present in the membranes of brain ECs, an observation made using transmission electron microscopy with immunogold labeling (Lu et al., 2019). Consistently, increased NO production in isolated mice ECs was observed in response to glutamate and D-serine application. In addition, activation of NMDARs caused dilation of isolated rat pial arteries (LeMaistre et al., 2012) and mouse arterioles in brain slices (Stobart et al., 2013). Moreover, endothelial NMDARs knockdown mice exhibited significantly reduced hyperemic responses to whisker stimulation in the barrel cortex (Hogan-Cann et al., 2019). Therefore, glutamate released by neurons or astrocytes could directly cause vasodilation of small arteries through stimulation of NMDARs on the cerebrovascular EC.

NMDARs in mouse brain microvascular endothelial cells (BMECs) are assembled of GluN1, GluN2A and GluN3A subunits. These receptors not only generate currents but also induce Ca^2+^ signaling in response to glycine treatment alone. As a result, glycine lowers the BBB integrity and compromises BMEC’s migratory capacity by reorganizing the actin cytoskeleton (Epping et al., 2022). There are several mechanisms underlying the increased permeability of the BBB following NMDAR activation in ECs including enhanced localization of clathrin heavy chain at the plasma membrane and aggregation of Caveolin-1. These changes trigger the activation of endosomal trafficking pathways. Thus, increased transendothelial transport in the presence of NMDA was confirmed for IgG and transferrin molecules. At the same time, knockout mice lacking NMDAR subunit NR1 in ECs exhibited reduced vascularization and a decreased number of neurons in the brain, with these deficits progressing in a time-dependent fashion after birth (Kim K.-S. et al., 2022). NMDARs in endothelial cells, in addition to regulating blood flow by endothelial NO synthase activation and NO release, and permeability of the BBB, may also stimulate generation of reactive oxygen species (ROS) (Sharp et al., 2005) and foster homocysteine-induced mitochondrial toxicity (Kamat et al., 2015).

It has also been shown that the primary cultures of BMECs contain alpha-amino-3-hydroxy-5-methylisoxazole-4-propionate receptor (AMPAR) subunits GluA1 (Andras et al., 2007) and treatment of BMECs with the AMPAR inhibitor 6,7-dinitroquinoxaline-2.3-dione (DNQX) prevents hypophosphorylation of threonine residues in the junction protein occludin caused by exposure to glutamate. In addition to ionotropic glutamate receptors, ECs express metabotropic glutamate receptors (mGluRs). Thus, it has been demonstrated that the brain microvascular EC line hCMEC/D3, a common model of the human BBB, expresses mGluR1 and mGluR5 (Negri et al., 2020). Ca^2+^ response to glutamate in this cell line was inhibited by pharmacological blockers of mGluRs, CPCCOEt and MTEP hydrochloride. Additionally, it has been confirmed that activation of mGluR stimulates a significant release of D-serine from astrocytes. D-serine acts as a mediator of neurovascular coupling, facilitating astrocyte-induced dilation of penetrating cortical arterioles. This effect depends on the availability of both extracellular glutamate and NMDARs (Stobart et al., 2013). The types of glutamate receptors present in endothelial cells are summarized in Table 1.

**TABLE 1:** Types of glutamate receptors expressed in brain endothelial cells.

Type of receptor	Subunits of receptor	Type of cell	References
NMDA receptor	NR1, NR2A–C	Cultured rat cerebral endothelial cells	Krizbai et al. (1998)
NMDA receptor	NMDAR-NR-1, NMDA-2B	Rat primary cultures of brain microvascular endothelial cells	Andras I.E. et al. (2007)
NMDA receptor	NMDAR1, NMDAR2A/B	Human brain endothelial cells	Sharp C.D. et al. (2003)
NMDA receptor	NR1	Human primary brain endothelial cells	Kim K.S. et al. (2022)
NMDA receptor	GluN1, GluN2C, GluN3B, GluN3C	Human hCMEC/D3 cell line	Negri S. et al. (2021b)
NMDA receptor	NDMAR1	Human retinal endothelial cells	Tawfik A. et al. (2021)
NMDA receptor	NMDAR1	Mouse brain endothelial cells culture b.End3	Kamat P.K. et al. (2015)
NMDA receptor	NR1	Endothelial cells isolated from male CD1 mouse brains	LeMaistre J. L. et al. (2012)
NMDA receptor	NR1, NR2A, NR2B, NR2C, NR3A, NR3B	Murine brain endothelial cell line cerebEND	Neuhaus W. et al. (2012)
NMDA receptor	GluN1, GluN2C	Mice brain endothelial cell cultures	Lu L. et al. (2019)
NMDA receptor	NMDA R1, NMDA R2A/B	SV129 mouse brain endothelial cell line b.End3	Scott G.C. et al. (2007)
NMDA receptor	GluN1, GluN2B, GluN3A	Mouse spinal cord vessels endothelial cells	Mehra A. et al. (2020)
NMDA receptor	GluN1, GluN2A, GluN3A	Mouse brain microvascular endothelial cells	Epping L. et al. (2022)
NMDA receptor	NR1, GluR1	Cerebral microvascular endothelial cells from newborn pigs	Parfenova H. et al. (2003)
Metabotropic mGluR	mGluR1alpha	Cerebral microvascular endothelial cells from newborn pigs	Parfenova H. et al. (2003)
Metabotropic mGluR	mGluR1, mGluR5	Human brain endothelial cell hCMEC/D3	Negri S. et al. (2020)
AMPA receptor	GluR1–4	Cultured rat cerebral endothelial cells	Krizbai et al. (1998)
AMPA receptor	GluR-1	Rat primary cultures of brain microvascular endothelial cells	Andras I.E. et al. (2007)

### Endothelium glutamate receptors in brain ischemia and hypoxia

Vascular pathology associated with aging and common chronic diseases, such as arterial hypertension, atherosclerosis and diabetes mellitus, is a leading cause of central nervous system dysfunction. Microvascular changes in small arterioles and capillaries lead to ischemia and hypoxia, which impair ATP production and reduce the ability of brain cells to remove glutamate from the extracellular space. As a result, the concentration of glutamate in the interstitial fluid increases considerably during transient cerebral ischemia (Ritz et al., 2004) and cerebral artery occlusion (Umemura et al., 1996), playing a key role in the development of ischemic brain damage (Huang et al., 2023). High extracellular glutamate levels can also lead to the disruption of tight junctions between ECs, leading to increased BBB permeability (Mehra et al., 2020) and promoting the development of edema, which can become more severe 24–72 h after ischemic stroke (Preston and Webster, 2004). Disruption of the BBB may also be enhanced by glutamine, which is released from neurons following brain injury and stimulates NMDARs causing increased influx of Ca^2+^ (Unterberg et al., 2004).

An increase in the BBB permeability due to activation of endothelial NMDARs is a critical pathogenic event in the development of ischemic stroke, as ischemia of the brain leads to development of edema and its severity is directly proportional to the area of ischemic lesion, the size of penumbra and the severity of stroke (Yang et al., 2019). Ischemia leads to generation of free radicals, neuronal damage and calcium efflux from the injured cells resulting in activation of the metabotropic calcium-dependent receptors, which activate NO synthase type 1. The latter causes exacerbation of edema and formation of metabolites, such as NO and superoxide anion, which interact to form the highly toxic peroxynitrite (Love, 1999). Glutamate efflux in the acute phase of ischemic stroke evokes excitotoxicity by excessive stimulation of NMDARs and AMPARs, which leads to enhanced calcium, water, and sodium influx into neurons, thus activating intracellular hydrolases and triggering neuronal death (Saha et al., 2021). Activation of glutamate receptors evokes changes in the proteomic profile of cerebral ECs (Minagar et al., 2009). Stimulation of NMDAR may also promote ferroptosis (regulated form of cell death attributed to abundant cellular iron levels that imbalance the production and clearance of lipid peroxides) of ECs (Han et al., 2024; Dixon et al., 2012). It has been found that triggering NMDARs by L-glutamic acid or NMDA in human umbilical vein ECs launches ferroptosis by elevating iron content, which in turn leads to accumulation of lipid peroxidation product malonic dialdehyde (MDA), increased expression of prostaglandin-endoperoxide synthase 2 (PTGS2), and depletion of the antioxidative enzyme glutathione peroxidase 4 (GPX4). NMDAR stimulation first causes activation of protein phosphatase 2 (PP2A), then dephosphorylation of AMP-activated protein kinase (AMPK), and finally increases the expression of protein HMGB1, a nucleus-located cytokin-like mediator of inflammation that sets off ferroptosis.

Necrosis, together with ferroptosis, in the tissue surrounding brain infarction and release of various DAMPs triggers an inflammatory response leading to the massive secretion of cytokines. In BMECs isolated from the brain cortex, the NMDAR channel blocker memantine (Sobolevsky and Koshelev, 1998; Parsons et al., 1993; Chen et al., 1992) decreased, while the agonist glycine increased the production of TNF-α, IL-6, IL-8, and IL-10 (Guan and Wang, 2023). In addition, expression of adhesion molecules ELAM-1, VCAM-1, and ICAM-1 on the surface of BMECs was down-modulated by memantine and up-modulated by glycine.

One more mechanism leading to NMDAR activation in ischemic stroke is the rapid release of the serine proteinase tissue-type plasminogen activator (tPA) from ECs, which also triggers vasodilation and exacerbates excitotoxicity (Yepes, 2024). It has been demonstrated that tPA released into circulation by the liver ECs reaches the brain and promotes functional hyperemia through activation of endothelial NMDARs (Furon et al., 2023). In mice, hyperemia evoked by whisker stimulation was significantly diminished in tPA knockdown line and increased in animals with deficient plasminogen activator inhibitor 1 (PAI-1). The mechanism by which tPA affects NMDARs is believed to involve stimulation of Ca^2+^ influx via direct interaction with the extracellular domain of GluN1 subunit (Nicole et al., 2001). An alternative is that tPA first binds to LRP1 (low density lipoprotein related protein-1) (Mantuano et al., 2013) and then modulates NMDAR function indirectly through the adaptor protein postsynaptic scaffold protein-95 (PSD-95) (Nakajima et al., 2013). While this mechanism was proposed for neurons, it might also be applicable to ECs. It has been shown that stimulation of type I metabotropic mGluR by glutamate in hippocampal neurons increases tPA synthesis by translational activation of the tPA mRNA (Shin et al., 2004). Therefore, glutamate plays a dual role turning on the NMDARs signal in EC with the tPA assistance and, on the other hand, enhancing the tPA synthesis and secretion by neurons through metabotropic mGluR.

As has been set out before, activation of NMDARs leads not only to vasodilation but also to increased permeability of the vessel wall. This effect has been demonstrated in mouse CNS ECs by stimulation of NMDARs with glutamate and glycine (Mehra et al., 2020). However, the permeabilization response of NMDARs in this case required coactivation by tPA. Moreover, the combined stimulation of NMDARs by its agonists and tPA generated a metabotropic signal instead of the ionotropic one (Ca^2+^ influx), likely due to the presence of subunit GluN3A as a part of the tetrameric receptor assembly. It was also shown that the metabotropic response in the presence of tPA was mediated by the Rho/ROCK pathway leading to phosphorylation of myosin light chain and reorganization of EC increasing the intercellular gaps.

Glutamate receptor mGluR1, together with NMDARs, may play a deleterious role in brain ischemia. Thus, YM-202074, a high-affinity antagonist of mGluR1, improved neurological deficit and reduced the infarct area in the hemisphere and cortex of rats after temporary occlusion of middle cerebral artery (Kohara et al., 2008). Similarly, RGS5 (regulator of G-protein signaling 5), a GTP-ase known as a negative regulator of the Gαq and Gαi subunits of G-protein coupled to mGluR1 (Fu et al., 2004), prevented elevation of permeability and maintained the integrity of the BBB in focal cerebral ischemia (Sladojevic et al., 2020). RGS5 inhibited ROCK and MLCK signaling pathways, causing actin cytoskeletal reorganization and affecting endothelial tight junction and stroke severity.

NMDARs and mGluR1 may share a common mechanism of increased BBB permeability mediated by the increased concentration of intracellular Ca^2+^. Glutamate can directly stimulate calcium influx via ionotropic NMDARs, while activation of mGluR1 can first lead to upregulation of phospholipase C and then generation of inositol triphosphate from phosphatidylinositol 4,5-biphosphate (Willard and Koochekpour, 2013). Inositol triphosphate regulates the exit of calcium from the endoplasmic reticulum. Elevation of intracellular calcium activates MLCK and phosphorylates MLC (Speyer et al., 2014; Legros et al., 2009), preceding the synthesis of actomyosin, contraction of ECs and rearrangement of tight junction proteins.

The loss of tight junctions between ECs is a hallmark pathologic event in many CNS disorders, including ischemia and stroke. Another mechanism of the BBB disruption was revealed in studies of BMEC primary cultures, where transient glutamate led to a decrease in the total amount of the tight junction protein occludin and its reduced localization in the cell–cell border segments, deteriorating the BBB barrier function (Andras et al., 2007). The redistribution of occludin was partially blocked by the NMDAR and AMPAR inhibitors MK-801 and DNQX, respectively. MK-801, but not DNQX, also prevented the glutamate-induced loss of the barrier function in co-cultures of BMECs and astrocytes. The effect of glutamate on redistribution of occludin in BMECs is associated with a receptor-mediated activation of downstream kinases. Signaling through NMDARs induces hyperphosphorylation of occludin’s tyrosines and hypophosphorylation of its threonines. Notably, incubation of cultured cerebral ECs with 2 mM glutamate increased phosphorylation of calcium/calmodulin-dependent protein kinase II (Krizbai et al., 1998). At the same time, inhibition of AMPARs/KARs by DNQX abolished hypophosphorylation of occludin’s threonine but did not stop the disintegration of the BBB. These data suggest that NMDARs and AMPARs/KARs control different signaling pathways involved in maintenance of the BBB integrity.

Stroke correlates positively with the elevated plasma levels of homocysteine (homo-Cys) (Smith and Refsum, 2021). Homo-Cys binds to the glutamate sites of NMDARs with high affinity (Doronzo et al., 2010). The corresponding activation of NMDARs by homo-Cys has been found to induce mitochondrial toxicity in ECs, which is manifested by accumulation of intracellular Ca^2+^, increased expression of H_2_O_2_-generating NADPH-oxidase-4 (NOX-4), and reduced levels of nitrite and superoxide dismutase (SOD-2) protein (Kamat et al., 2015). Moreover, homo-Cys reduces the mitochondria membrane potential and ATP production. H_2_S and NMDAR siRNA-treated ECs show decreased mitochondrial toxicity. H_2_S may have a protective effect through epigenetic changes, because incubation of ECs with homo-Cys in the presence of DNA methyltransferase inhibitor 5′-azacitidine annihilated the deleterious effects of the former. Thus, homo-Cys may incur an additional pathogenic effect on BMEC permeability in stroke patients.

**FIGURE 1:**
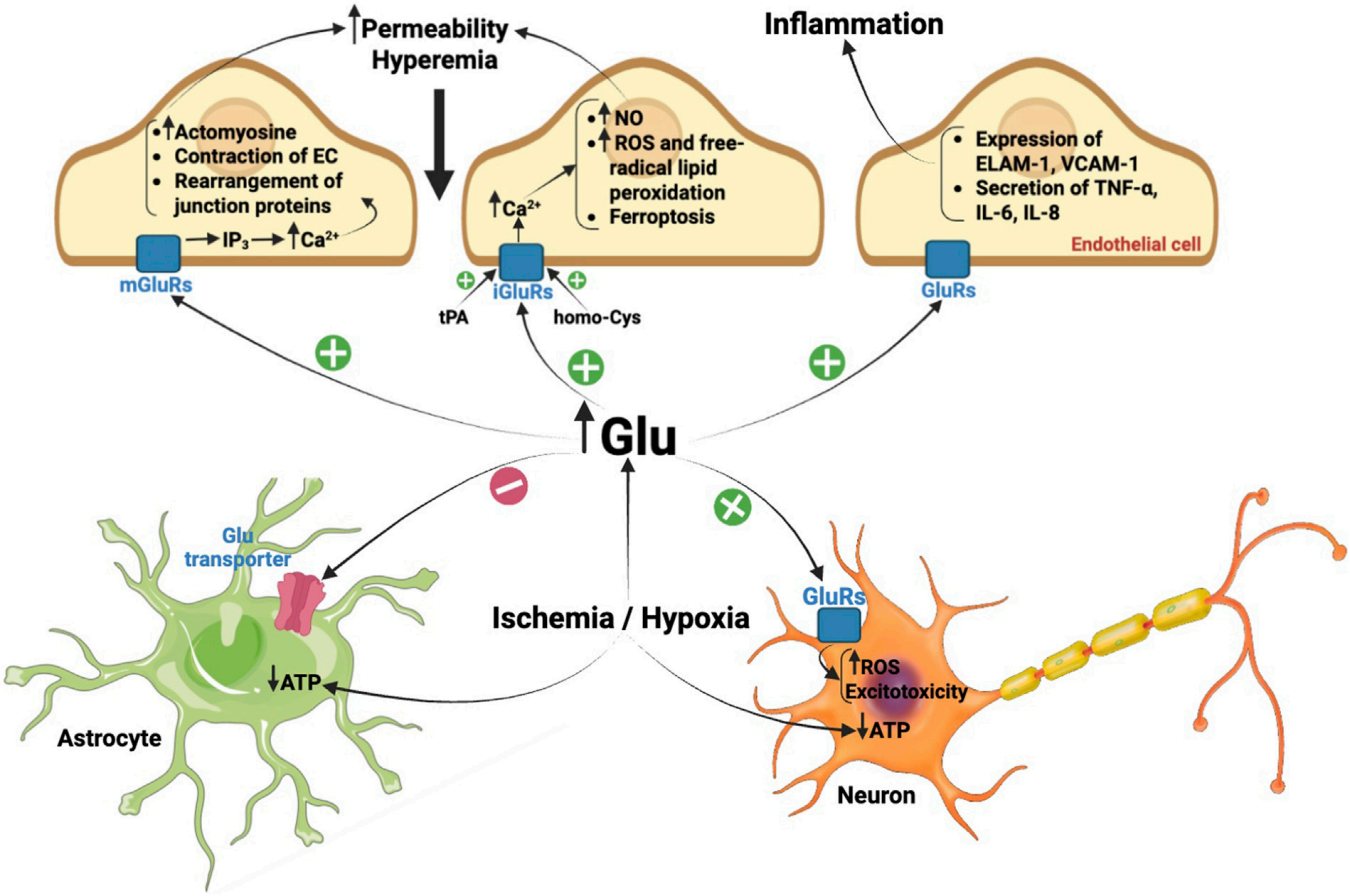
Mechanisms of ischemic/hypoxic brain injury that involve endothelial glutamate receptors. Ischemia and hypoxia lead to low rates of ATP synthesis which limit the ability of astrocytes to remove glutamate from extracellular space. As a result, concentration of glutamate in the interstitial fluid considerably increases. Glutamate stimulates the inward Ca^2+^ flow by activation of ionotropic and metabotropic mGluR1 glutamate receptors in ECs. Stimulation of NMDAR can launch ferroptosis due to intracellular accumulation of free iron and activation of free-radical lipid peroxidation. Agonists of glutamate receptors cause a decrease in the total amount of tight junction protein occludin and reduce its distribution at cell–cell border segments increasing the permeability of the endothelial layer. Accumulation of glutamate in the brain tissue and excessive stimulation of NMDA and AMPA receptors evokes excitotoxicity, which triggers neuronal death and inflammatory response. Glycine and other NMDAR agonists increase production of TNF-α, IL-6, IL-8, and IL-10, and expression of the adhesion molecules ELAM-1, VCAM-1, and ICAM-1 on the surface of ECs enhancing inflammation. Activation of endothelial NMDARs is potentiated by increased concentrations of tPA and homocysteine in blood plasma. Decryption of symbols: ATP–adenosine triphosphate, ELAM-1 – endothelial leukocyte adhesion molecule 1, GluRs–glutamate receptors, iGluRs–ionotropic glutamate receptors, mGluRs–metabotropic glutamate receptors, homo-Cys–homocysteine, ICAM-1 – inter-cellular adhesion molecule 1, IL–interleukin, IP_3_ – inositol triphosphate, NO - nitric oxide, ROS–reactive oxygen species, TNF-α–tumor necrosis factor alpha, tPA–tissue-type plasminogen activator, VCAM-1 – vascular cell adhesion molecule 1.

The mechanisms of ischemic/hypoxic brain injury which involve endothelial glutamate receptors are presented in Figure 1.

### Endothelium glutamate receptors in traumatic brain injury (TBI)

TBI is one of the leading causes of injury-related death and disability worldwide. The pathogenesis of TBI is primarily determined by injury of brain cells and neuroinflammation, which is directed by local microglia and ECs, and migrating neutrophils (Cash and Theus, 2020). BMECs form the first barrier that neutrophils must cross to reach the site of injury. An experimental model of TBI has been used to demonstrate the role of metabotropic glutamate receptors mGluR5 in regulating the transmigration of neutrophils. Following brain trauma, the integrity of the BBB is significantly higher, and the extent of the neutrophils infiltration is much lower in mGluR5 knockout (KO) mice compared to the wild-type (WT) littermates (Yang et al., 2017). Moreover, knockout of mGluR5 reduces the mRNA levels of endothelium generated chemokines, including CXCL1, CXCL2, CCL2, CCL4 and CCL5, which attract neutrophils to the site of the brain tissue inflammation. Furthermore, neurological dysfunction scores were lower in mGluR5 KO compared to WT mice in the acute phase of TBI. Therefore, blocking mGluR5 receptors in the ECs may have a protective significance in TBI.

mGluRs may contribute to the maintenance of the neuroinflammatory process in TBI through astrocytes. It has been shown a mGluR agonist induced a significant release of D-serine from astrocytes (Stobart et al., 2013). D-serine then binds to NMDARs in ECs, inducing dilation of cortical arterioles in the presence of the excess glutamate.

### Endothelium glutamate receptors in Alzheimer’s disease (AD)

AD is a slowly progressing neurodegenerative disease characterized by the deposition of A_β_-containing plaques in the interstitial space and the formation of aggregates of hyperphosphorylated tau-protein (neurofibrillary tangles, NFT) in neurons of specific brain regions. Both A_β_ and NFT are believed to cause significant neural dysfunction, leading to dystrophy, cell death and neuroinflammation (Conway, 2020).

Abnormal BBB permeability is believed to play a crucial role in AD pathogenesis. At early stages of AD, there is a significant breakdown of the BBB, which coincides with the cognitive decline, and reduction in the blood flow in familial AD after deposition of A_β_ and before the onset of clinical manifestations (Fisher et al., 2022). The A_β_(1-40) peptide, which aggregates to form amyloid during AD, has been found in perivascular space and, in about 80% of cases, in the walls of cerebral blood vessels (Ellis et al., 1996). Recordings of Ca^2+^ efflux events in ECs showed that A_β_(1-40) inhibits NMDAR function, manifested as a suppression of NMDAR-elicited Ca^2+^ signals and reduced dilation of the cerebral artery (Peters et al., 2022). In experiments with isolated posterior communicating arteries from 5x-FAD mice, a transgenic model of the early-onset Alzheimer’s disease, the vasodilatory response to NMDA exposure was significantly inhibited compared to the wild-type counterparts. In addition, A_β_ plaques were observed around pial arteries and arterioles, and 45% of pial arteries from 5x-FAD mice demonstrated perivascular A_β_ accumulation.

Elevated plasma plasminogen activator inhibitor-1 (PAI-1) levels have been observed in individuals with mild cognitive impairment and patients with AD (Oh et al., 2014). This elevation is also noted in the Tg2576 mouse model of AD, which carries a mutation in the amyloid precursor protein (APP) (Park et al., 2020). Increased levels of PAI-1 may compromise the vasodilatory effects of tPA (mediated through NMDAR in endothelial cells), exacerbating neurovascular dysfunction and promoting neuroinflammation, which are hallmark features of Alzheimer’s pathology. Elevated PAI-1 levels could therefore be a potential biomarker for neurovascular damage in AD and may serve as a target for therapeutic intervention to restore the BBB integrity and reduce neuroinflammation. Indeed, the plasminogen activator inhibitor-1 (PAI-1) inhibits the tissue plasminogen activator (tPA), which is essential for facilitating neurovascular hyperemia, a process in which the blood flow is increased in the brain regions with heightened neuronal activity. This response is crucial for maintaining the proper brain function and supporting NVC (Park et al., 2020). Thus, the deficit of tPA reduces functional arterial hyperemia due to insufficient stimulation of NMDARs and diminished NO synthesis, while the exogenous tPA or its increased activity in tg2576 carrier mice prevent the mitigation of blood flow increase elicited by whisker stimulation. Additionally, the increased tPA activity improved cognition in 11–12-month-old tg2576 mice.

A cross-sectional study of 456 individuals with dementia revealed that patients with AD have increased blood levels of homo-Cys (Moretti et al., 2017). Moreover, high plasma levels of homo-Cys appear as a strong, independent risk factor for dementia in patients with AD (Seshadri et al., 2002). Accumulation of homo-Cys in blood of AD patients causes EC injury due to an NMDAR-mediated increase in intracellular Ca^2+^ and deterioration of mitochondria (Kamat et al., 2015). Thus, the build-up of metabolites in plasma, such as homo-Cys, or excessive generation of PAI-1 early during AD promote NMDAR-associated ECs dysfunction, impair NVC and increase BBB permeability. In this context, tPA plays a key role in NMDAR activation in ECs. NMDAR activation is critical for processes like vasodilation and blood flow regulation during neuronal activity. When tPA is inhibited by PAI-1, activation of NMDARs is impaired, causing NVC disruption and limiting the brain’s ability to increase blood flow in response to neuronal demand. As a result, increased levels of PAI-1 can reduce the ability to properly modulate the brain blood flow, which is particularly problematic in neurodegenerative conditions, like Alzheimer’s disease, where neurovascular dysfunction is common. Thus, PAI-1 not only affects fibrinolysis but also impairs the neurovascular response, which supports brain function, highlighting its potential as a biomarker and also as a therapeutic target in neurological diseases. These findings suggest that tPA may have broader therapeutic implications, not only for its role in clot breakdown, but also as a potential modulator of neurovascular health and cognitive function. By restoring the proper NVC and improving blood flow, tPA may help mitigate some of the vascular and cognitive impairments associated with Alzheimer’s and other neurodegenerative diseases.

### Endothelium glutamate receptors in autoimmune diseases that affect the brain

Systemic lupus erythematosus (SLE) is a chronic autoimmune disease characterized by production of autoantibodies against a wide range of self-antigens, including the components of DNA/protein and RNA/protein complexes, phospholipids, histones, ribosomes and brain glutamate receptors. The assembled immune complexes circulate in the bloodstream and then gradually penetrate the wall of small arterioles in many organs, where they initiate an inflammatory process in accordance with the type III hypersensitivity reaction. Patients with SLE often develop pathology of the central nervous system referred to as neuropsychiatric SLE. This condition is characterized by headache and cognitive disorders but may also be accompanied by memory loss, anxiety and depressive mood (Recio-Barbero et al., 2024; Schwartz et al., 2019).

In the study on MRL/lpr mice, an animal model of SLE, antibody levels against NMDAR subunits NR2A/B (anti-NR2A/B) were significantly increased compared to the MRL/MPJ mice, their control counterparts (Guan and Wang, 2023). In addition, a series of tests showed that MRL/lpr mice behaved in a depressive manner and demonstrated reduced locomotion/exploratory activity, high levels of anxiety and poor learning/memory capability. The NMDAR channel blocker memantine stimulated the proliferation of the isolated brain cortex microvascular endothelial cells (MVEC), whereas its agonist glycine had the opposite effect. At the same time, memantine reduced while glycine increased secretion of inflammatory mediators TNF-α, IL-6, IL-8, and IL-10 by MVEC from the MRL/MPJ mice. Similarly, memantine downregulated, while and glycine upregulated the expression of adhesion molecules ELAM-1, VCAM-1, and ICAM-1 in MVEC. The anti-NR2a/2b antibody exerted the same effect on the adhesion molecules as glycine. The results of these experiments suggest that modulation of endothelial NMDARs by agonists or antagonists can help to control synthesis of the inflammatory mediators and adhesion molecules, ultimately reducing the intensity of neuroinflammation. This approach offers a potential strategy for mitigating symptoms of anxiety, depression and cognitive impairment in patients with neuropsychiatric SLE.

Multiple sclerosis (MS) is a demyelinating CNS disease. Its pathogenesis complies with the type IV hypersensitivity reaction and involves immune autoaggression of CD4^+^ Th_1_ and Th_17_ cells against the components of the myelin sheath, such as myelin basic protein, proteolipid protein and myelin oligodendrocyte glycoprotein. CD8^+^ T-lymphocytes and B-lymphocytes also play a role in the pathologic process resulting in inflammation, myelin injury, microglia and astrocyte activation (gliosis) and loss of neurons (da Silva et al., 2020). BBB permeability increases in the early stages of MS, promoting inflammation and local accumulation of mononuclear cells, which leads to demyelination and injury of axons. The brain ECs appear to express NMDAR subunit NR1 at the cell-cell junctions, close to protein ZO-1 (Reijerkerk et al., 2010). In the same study, NMDAR activation was also shown to increase the passage of monocytes across the ECs layer, a process that is potentiated by tPA via NR1 subunit binding site. The role of NMDARs in increasing BBB permeability in MS was further supported by the effects of a mononuclear antibody against the tPA-sensitive site on NR1 (Macrez et al., 2016). The study found that this antibody inhibited transmigration of monocytes and T-lymphocytes through the monolayer of brain ECs *in vitro*.

Anti-N-methyl-D-aspartate receptor encephalitis is an autoimmune disease characterized by behavioral disorders, memory impairment, psychosis and seizures (Seery et al., 2022). Anti-NMDAR antibodies circulate in blood, cross the BBB and bind to GluN1 subunit on the surface of neurons and astrocytes, where they induce internalization of the target receptors (Ismail and Faustmann, 2020). Anti-NMDAR antibodies also trigger an inflammatory response in the brain tissue (encephalitis), though the immediate phlogogenic factor remains unclear. The ultimate result of this pathologic process is impairment of higher nervous functions. Permeation of the brain tissue by anti-NMDAR antibodies is facilitated by increased permeability of the BBB. In experiments with the humanized mouse model of anti-NMDAR encephalitis, human antibodies increased extravasation of fibrinogen, indicating elevation of BBB porosity, possibly due to the increased production of IL-1β by ECs (Shu et al., 2023). Although these studies did not verify the interaction of antibodies with EC NMDARs, IL-1β secretion was presumably enhanced by the Ab-receptor binding, as it has been demonstrated before for various inflammatory mediators and adhesion molecules in isolated cells of the SLE model (Guan and Wang, 2023).

### Development of potential therapeutics targeting endothelial glutamate receptors

Glutamate receptors have been proposed as potential therapeutic targets for a variety of neurodegenerative diseases, as well as ischemia and TBI. In this review we focus specifically on potential therapeutics aimed at regulation of the BBB through the effects on endothelial glutamate receptors.

A monoclonal antibody Glunomab was designed to bind to a tPA-sensitive regulatory site on the GluN1 subunit of NMDAR. It has been shown that Glunomab binds to NMDAR at the luminal surface of spinal cord ECs *in vivo* (Macrez et al., 2016). When injected to animal models of experimental autoimmune encephalomyelitis, this antibody reduced demyelination at the loci of inflammatory lesions and prevented progression of neurological deficit. *In vitro* experiments with the BBB model of human cerebral ECs showed that Glunomab reduces the transmigration of human monocytes and T-lymphocytes stimulated by TNF (inflammatory conditions). Thus, the antibody targeting NMDARs appeared to be efficient in restoring the blood-spinal cord barrier and preventing the local accumulation of autoaggressive immune cells.

Another therapeutic agent that shows promise in regulating BBB permeability is perampanel, a noncompetitive antagonist of AMPA and kainate receptors that binds at the ion channel extracellular collar (Gangwar et al., 2023a, Gangwar et al., 2023b; Yelshanskaya et al., 2016; Hibi et al., 2012). Perampanel protected the NVU system (neurons, astrocytes, and BMECs) against traumatic and excitotoxic (100 μM glutamate) injury *in vitro* and in modeling TBI *in vivo* (Chen et al., 2021). Beyond preserving the BBB integrity, perampanel suppressed lipid peroxidation and exhibited anti-inflammatory activity that reduced the secretion of IL-1β and IL-6 in the NVU system. Additionally, it diminished apoptosis in the brain tissue after TBI. These protective effects of perampanel were linked to the increased expression of the mitochondrial histone deacetylase Sirt3. However, several side effects have been identified in patients with neurodegenerative diseases who were treated with perampanel (Vukolova et al., 2023).

Microvascular regulation of brain function through NVC involves not only dilation of small arterioles and increase in blood flow, but also transmural events, including activation of the paracellular pathway and transcytosis of large molecules across the layer of ECs. Alterations in the paracellular pathway involve the number and cellular distribution of adherence junctions and tight junction proteins (Keaney and Campbell, 2015). These changes are associated with actin-cytoskeletal reorganization driven by GTPases, such as RhoA (Sit and Manser, 2011). Transcytosis involves internalization and transport across the EC using endosomal trafficking pathway (Villaseñor et al., 2019).

*In vitro* experiments have shown that the increased NMDAR-mediated Ca^2+^ influx into human brain primary ECs resulted in redistribution of the clathrin heavy chains to the cell surface and aggregation of Caveolin-1 (Kim K.-S. et al., 2022). Both proteins are involved in internalization pathways that converge within the early endosome network, which carries sorting function (Villaseñor et al., 2019). Moreover, stimulation of NMDARs increased the transcytosis by elevating the number of early endosomes and reducing the extent of lysosome trafficking. These effects were shown to be mediated through activation of calmodulin-dependent protein kinase II (CaMKII) and RhoA/protein kinase C. Consistent with the *in vitro* results, administration of NMDA to mice stimulated the transendothelial delivery of IgG and transferrin molecules, which are typically transported across the BBB at lower rates. Since some therapeutic measures may require a temporary increase in BBB permeability to facilitate drug delivery in the brain regions, development of factors that facilitate para- and transcellular transfer of molecules may have significant practical value.

## Conclusion

The loss of the endothelial layer integrity has been identified as a critical factor contributing to the increased BBB permeability and plays a key role in the pathogenesis of various neurological disorders, including Alzheimer’s disease, autoimmune brain injury, Parkinson’s disease, amyotrophic lateral sclerosis, and acute ischemic CNS injury. Dysfunction of BMECs leads to extensive penetration of the BBB and accumulation of pathological proteins, such as Aβ or autoantibodies, in the brain tissue, resulting in neuronal dystrophy and apoptosis, and initiating neuroinflammation. Activated BMECs can themselves become the source of proinflammatory mediators. Based on this concept, gaining a deeper understanding of the mechanisms of regulation of the BBB function is essential to develop new strategies for the treatment of cerebrovascular and neurological diseases. In this review, we focused on the evidence of the glutamate receptors-mediated alteration of endothelial permeability and potential for the brain pathology prevention by direct effects on NMDA and AMPA receptors. We have not covered other mechanisms of the BBB modification, such as inhibition of the PDGF-C/PDGFR-α signaling axis (Matsuoka et al., 2022). Therefore, further work is needed to illuminate the mechanisms of molecular transfer through the BBB and to develop effective BBB-targeted treatment approaches for neurological diseases.
